# Water friction in nanofluidic channels made from two-dimensional crystals

**DOI:** 10.1038/s41467-021-23325-3

**Published:** 2021-05-25

**Authors:** Ashok Keerthi, Solleti Goutham, Yi You, Pawin Iamprasertkun, Robert A. W. Dryfe, Andre K. Geim, Boya Radha

**Affiliations:** 1 Department of Chemistry, University of Manchester, Manchester, UK; 2 National Graphene Institute, University of Manchester, Manchester, UK; 3 Department of Physics and Astronomy, University of Manchester, Manchester, UK; 4 Present Address: Faculty of Sciences and Liberal Arts, Department of Applied Physics, Rajamangala University of Technology Isan, Nakhon Ratchasima, Thailand

**Keywords:** Surfaces, interfaces and thin films, Mechanical and structural properties and devices, Nanofluidics, Two-dimensional materials

## Abstract

Membrane-based applications such as osmotic power generation, desalination and molecular separation would benefit from decreasing water friction in nanoscale channels. However, mechanisms that allow fast water flows are not fully understood yet. Here we report angstrom-scale capillaries made from atomically flat crystals and study the effect of confining walls’ material on water friction. A massive difference is observed between channels made from isostructural graphite and hexagonal boron nitride, which is attributed to different electrostatic and chemical interactions at the solid-liquid interface. Using precision microgravimetry and ion streaming measurements, we evaluate the slip length, a measure of water friction, and investigate its possible links with electrical conductivity, wettability, surface charge and polarity of the confining walls. We also show that water friction can be controlled using hybrid capillaries with different slip lengths at opposing walls. The reported advances extend nanofluidics’ toolkit for designing smart membranes and mimicking manifold machinery of biological channels.

## Abstract (web summary)

Flow through nanometer scale channels facilitates an unmasked study of water-surface molecular interactions. Here, Keerthi et al. show with conduits made from graphite and hexagonal boron nitride that strong hydrophobicity does not rule out enhanced stickiness and friction.

## Introduction

Water conduction through porous materials and membranes plays a significant role in many natural and artificial phenomena such as molecular transport in biological cells, osmotic power generation, membrane-based molecular separation, as well as in water purification technologies^1–5^. Inspired by the fast and selective water flow through protein channels (e.g., aquaporins), several artificial systems have been suggested to mimic them^1,2,6,7^. Central to the functioning of such protein channels are appendant surface functional groups and, importantly, Å-scale confinement^1^. Early on, carbon nanotubes were the imminent fluidic conduits^6,8–10^, thanks to their one-dimensional architecture permitting a single-file water flow, similar to aquaporins. Over the last decade, quasi-zero dimensional pores and two-dimensional (2D) channels made from a variety of 2D materials have been reported^11,12^. It has been confirmed in many studies that water flow through channels with dimensions below a few nanometers can display a drastic enhancement. However, a majority of these studies were using carbon-based surfaces (e.g., carbon nanotubes^6,7,9,10^, graphene^13^, and graphene oxide membranes^14–16^) with a few exceptions of other materials such as molybdenum disulfide (MoS_2_) and MXene membranes^17^, and hydrophilic alumina surfaces^18^. Hence, the flow enhancement was primarily attributed to frictionless transport of water along atomically flat graphitic surfaces^6–9^. Nevertheless, it remains unclear which factors and interactions contribute to the ultrafast water flow under confinement^19^ because of the lack of experimental systems made controllably from different materials but with similar dimensions. Theoretical studies have extensively discussed the ultrafast water transport, suggesting electrohydrodynamic coupling, electrostatics, etc. as possible reasons for low-friction flows^8,20,21^. In this report, we investigate the relationship between water–surface friction and the electronic, structural, and chemical properties of confining atomically flat materials such as graphene, hexagonal boron nitride (hBN), and MoS_2_, using the recently demonstrated Å-scale capillaries made from various 2D materials^22^.

## Results and discussions

### Å-capillary device fabrication

**Fig. 1:**
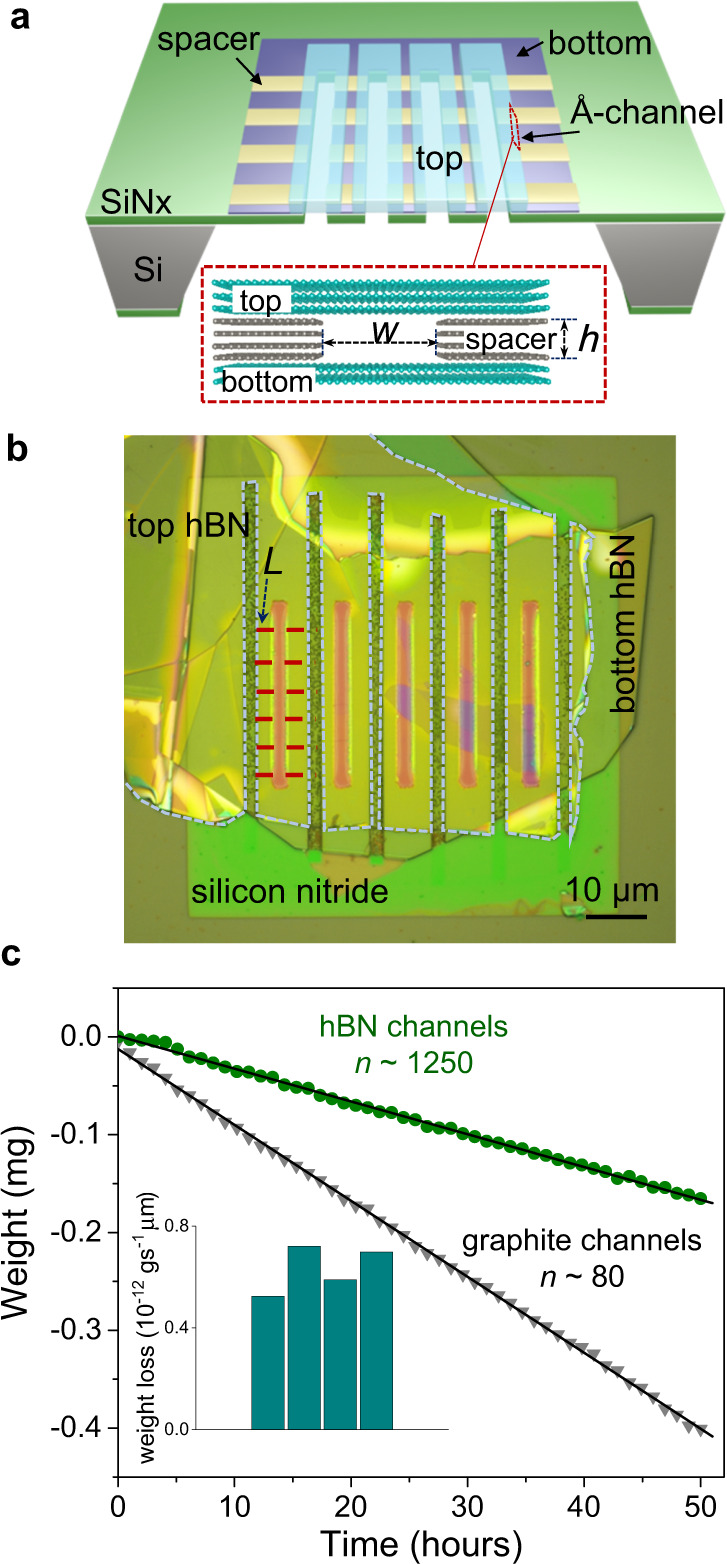
Water flow through graphite vs. hBN capillaries. **a** Schematics of our capillary devices. **b** Optical image of a hBN capillary device on a silicon nitride membrane (seen in light green). The top hBN layer is contoured with the dotted curve for visibility. Five parallel rectangular holes (light purple color) open to the other side of the wafer. The direction of the channels underneath the top hBN layer is indicated by the red dotted lines with a defined length *L*. **c** Weight loss due to water evaporation through four-layer (*h* ≈ 1.4 nm) capillaries. The hBN device (green circles) has ~1250 (±40) parallel channels and the flow is normalized for channel length of *L* ∼ 1 µm. The graphite device (grey triangles) has ~15 times fewer channels (*n* ≈ 80 (±4), average *L* ≈ 6 µm). The bottom inset shows the weight loss from four different hBN devices normalized per µm channel length. Schematics of water flow measurement setup and the details of monitoring the weight loss of water through the microgravimetry setup are in Supplementary Fig. 2.

The Å-capillary devices were fabricated following the previously described recipe with additional improvements^22^. The capillaries comprise three layers, namely bottom, spacer and top layers made from 2D crystals such as graphite, hBN, or MoS_2_, which are stacked using mechanical-transfer techniques (further details in Supplementary Fig. S1). The capillaries are supported by a free-standing silicon nitride membrane with pre-etched holes (Fig. 1a). A thin bottom layer (approx. 20 to 50 nm-thick) and a pre-patterned graphene spacer (number of graphene layers, *N* from 2 to about 25 layers) were transferred onto the holes in the silicon nitride membrane. The holes were projected into the bottom and spacer layer by dry etching, followed by sealing the assembly with a thick (approx. 100 to 150 nm) top layer. A post-processing lithography step defined the length of the capillaries to be about *L* ∼ 1 to <10 μm. The width of the capillaries is *w* ≈ 130 nm, whereas height *h* (=*N* × 0.34) ranges from *h* ∼ 0.68 nm to ∼8.5 nm (as indicated in Fig. 1a) and, in each device, the number of channels varies from *n* ∼ 80 to ∼2000 (see Supplementary Fig. 1 for further details).

### Measuring water flow through the 2D channels

To quantitatively study the water flow through Å-capillaries, we used microgravimetry (precision, 1 µg). A certain amount of water was placed in a miniature container that was sealed with the silicon chip containing the capillary devices, so that the only path for water permeation was via capillaries (Supplementary Fig. 2). As water evaporates through capillaries, there is a finite weight loss that was measured as a function of time.

As a reference, we measured devices made in exactly the same way but without graphene spacers. Even after several days of measurements, the control devices did not show any discernible water permeation apart from random drifts in the total weight measurements. As shown in Fig. 1c, the weight loss was linear in time for both graphite and hBN capillary devices that had spacers of height *h* ≈ 1.4 nm (*N* = 4) in height. However, the water loss differed greatly between them, by more than two orders of magnitude. In order to detect a water flow above our detection limit (approx. 5 × 10^−14^ g s^−1^), we had to increase the number of channels in hBN devices by at least 10–20 times, which was done by incorporating several parallel holes in the supporting silicon nitride membrane. With the capillaries of such a small size, there is a possibility that they could be clogged either during device fabrication or by hydrocarbon absorption, while storing them under ambient conditions. To crosscheck if this could be the reason for lower water flux observed in hBN capillaries, we performed gas flow measurements on the same devices before and after water permeation. The gas flows were observed to be similar in both graphite and hBN channels and were enhanced by over two orders of magnitude compared to expectations for the Knudsen gas transport^23^, which indicated that hBN capillaries were open and had a similar level of cleanliness as graphite capillaries.

### Water slip in hBN vs. graphite channels

**Fig. 2:**
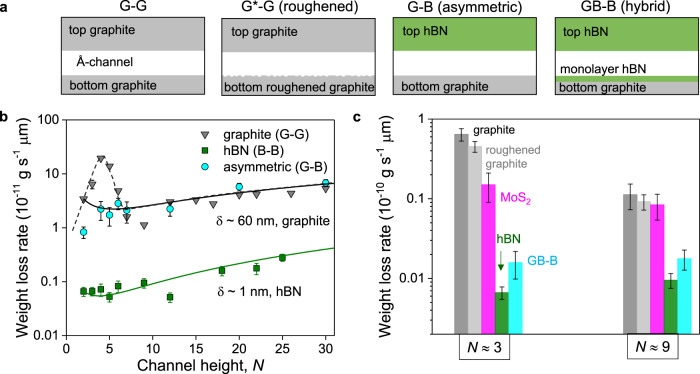
Influence of capillary walls’ material on evaporation. **a** Schematic of the devices with different wall materials’ combination. A hyphen is placed in the acronym to represent spacer (which is always graphene) present in between the bottom and top walls. As illustrated, device G-G (also referred as graphite channel) has both the bottom and top graphite walls. Similarly, hBN channel (B-B) has both the bottom and top walls made from hBN crystals. Asymmetric capillaries contain bottom and top walls made from different 2D materials (e.g., G-B has bottom graphite and top hBN). Hybrid capillaries refer to those where the bottom is contoured with another 2D material (GB-B has bottom graphite contoured with monolayer hBN and top hBN). **b** Water flow through graphite (grey triangles), hBN (green squares), and asymmetric G-B devices (cyan circles) with different channel heights. Results of hBN channels are compared with those for graphite channels reported in ref. ^22^. The weight loss is normalized for *L* ≈ 1 µm and per channel. The symbols represent the experimental data; the solid curves are the best fits using Eq. (1); the driving force is a combination of capillary and disjoining pressures. The fits yield the slip lengths *δ* ≈ 1 and *δ* ≈ 60 nm, for hBN and graphite, respectively. Dotted line is a guide to the eye, showing the deviation from the Eq. (1) for the sub-2 nm water flow rates. The anomalous peak at *N* ∼ 4, which deviates from the fitting, observed for graphite capillaries, is due to increased structural ordering of water. **c** Water flow rates for capillaries made of graphite (gray bar), roughened graphite G*-G (light grey bar), hBN (green bar), MoS_2_ (pink bar), and hybrid channels GB-B (cyan bar); spacers heights *N* = 3 and 9. Error bars in **b** and **c** indicate the data scatter for two devices measured for that particular channel height.

To understand the unexpectedly high flow rates of water through graphite capillaries compared to hBN ones, we measured several devices with different heights (Fig. 2). Both hBN and graphite capillaries show a non-monotonic increase with *N* and, clearly, graphite capillaries exhibited an order of magnitude higher fluxes than hBN devices, even for the largest *N* (Fig. 2b). Except for the walls’ material, all dimensions of the graphite and hBN capillaries, and measurement conditions were identical; therefore, similar water evaporation rates could be expected, in contrast to the experimental results. To understand the discrepancy, we use the classical hydrodynamics equation modified by the slip term, which gives the flux *Q* per single channel as^11^

1 $$Q={\rho }_{0}\frac{P}{12{\eta }}\frac{w}{L}{h}^{3}\left[1+\frac{6{\rm{\delta }}}{h}\right]$$

where *ρ*_0_ and *η* are the density and viscosity of water, respectively, *δ* is the slip length, and *P* is the driving pressure, which includes both capillary pressure and disjoining pressure contributions^22^, given as *P* = *P*_cap_ *+* *P*_disj_. The capillary pressure *P*_cap_ is associated with the curved meniscus at the water–solid interface, which is given by *P*_cap_ = 2*σ*cos*(ɸ)h*^−1^, where *σ* is the surface tension of the water and *ɸ* is the contact angle of water. The second contribution disjoining pressure *P*_disj_ is due to the water–surface interactions, which scale rapidly with confinement height. *P*_disj_ can be approximated with its van der Waals contributions as *A* (6*πh*^3^)^−1^, where *A* is the Hamaker constant for water–graphite interaction^24^ (*A* ≈ 115 zJ) and is taken as the same value for hBN. Fitting our experimental data in Fig. 2b with Eq. (1) yields *δ* ≈ 60 nm and *δ* ≈ 1 nm for graphite and hBN capillaries, respectively^25^. The obtained slip length values compare well with those reported in previous molecular dynamics simulation studies. For example, the slip length of water on graphitic surfaces was reported to be 60 ± 6 nm by Kannam et al.^26^ and as approx. 80 nm by Falk et al.^8^, whereas on hBN surface, *δ* was reported to be 3.3 ± 0.6 nm^27^, which are consistent with our findings. Several experimental studies have reported varied *δ* values, e.g., *δ* ∼ 8 nm was observed on graphite surface^28^ and a median *δ* ∼ 16 nm with a spread of values between 0 and 200 nm was obtained inside graphene channels with confinements ranging from 20 to 120 nm^13^. It has been acknowledged in literature that several factors can affect the experimentally reported slip length values. For example, synthesis route of 2D materials, molecular adsorption during storage, and measurement can also lead to varied surface charges^13^. For the hBN channels, the water slip length has been experimentally found to be <5 nm in a BN nanotube with a diameter of approx. 50 nm^7^, which falls in line with our obtained value of *δ* ∼ 1 nm for hBN channels.

Although Eq. (1) fits apparently well for *N* > 7, there is a pronounced deviation for thin capillaries, especially in the sub-2 nm graphite capillaries. This additional enhancement can be attributed to the increased structural order of water under strong confinement^22^, whereas such peak was not observed for hBN capillaries. We have considered bulk viscosity of water here for the curve fitting at all channel heights (*N)*. However, the structural ordering in water under atomic-scale confinement can lead to an increase in the water viscosity by a factor of two or more^29,30^. Due to the uncertainty in the position-dependent viscosity^29,30^ value along the channel height, we resort to using the bulk viscosity for curve fitting. The water velocity estimated inside the graphite capillaries reaches up to 1 m s^−1^, whereas it is only approx. 10^−2^ m s^−1^ for hBN capillaries (Supplementary Note 2). This enormous difference is intriguing, as both the graphite and hBN surfaces are atomically flat and isostructural, only with a minute lattice difference^31^ of approx. 1%. Numerical simulations have previously shown a difference in friction of water on hBN and graphite of about three times^27^. In our experiment, we observe a much larger difference, one to two orders of magnitude depending on the capillary height. To probe this further, we have made asymmetric capillaries where one surface was graphite and the other was hBN, and we studied water flows. Remarkably, the water flow was comparable to that in graphite capillaries (Fig. 2b), which cannot be explained solely by the standard slip flow analysis with one high-slip and another low-slip channel wall. Although the flow rates in asymmetric and graphite capillaries are similar, there was no anomalous peak at *N* ∼ 4 in asymmetric ones, as observed in graphite capillaries. To find possible reasons that can explain the observed differences in water flow through graphite and hBN capillaries, we examined their electronic and wetting properties, the surface charge, and electrostatic interactions, which can all likely cause pinning of water molecules.

### Effect of electronic property of channel wall’s material

Although graphite and hBN share a similar crystallographic structure, their electronic properties differ—graphite is relatively highly conducting, whereas hBN is a wide-gap insulator. To examine the possible effects of electrical conductivity of confining material on water flow, we compared water flows through capillaries made of graphite and hBN with those made from semiconducting MoS_2_ that was heavily doped^32,33^, making it electrically close to graphite. As shown in Fig. 2c for different channel heights (*N* = 3 and 9), MoS_2_ capillaries exhibited water flow rates approximately two to eight times lower than graphite capillaries but still considerably higher than that observed in hBN ones. As the MoS_2_ and graphite walls in our experiments had comparable conductivities, we also made a hybrid capillary where the bottom wall was graphite contoured by monolayer hBN (subsequently referred to as GB-B device). As monolayer hBN provides little electrostatic screening^34^, the GB-B capillaries should be similar electronically to the graphite ones, despite the presence of the hBN on surface. The measured water flow through the hybrid GB-B capillaries was found to be two to three times larger than that in B-B capillaries; however, it was rather much smaller than that in graphite capillaries. This observation rules out the possibility that electrical conductivity of the capillary walls governs water transport and indicates the importance of the immediate water–solid interface.

### Effect of wettability of channel wall’s material

We also studied the effect of surface-wetting properties on water flow. Freshly cleaved crystals of hBN, graphite, and MoS_2_ exhibit similar water contact angles ranging from approx. 60° to 75° but the contact angle rapidly (within minutes) increases under exposure to ambient air because of adsorption of airborne hydrocarbons^35–38^. Let us note that despite the polar nature of hBN with charge separation of boron and nitrogen atoms, the experimental contact angles were, on contrary, indicative of mildly hydrophobic nature. After an hour under ambient conditions, all the three materials exhibited similar contact angles of approx. 75° to 80°, which increased eventually to 85°–90° (Supplementary Fig. 5). Given similar wetting properties of the capillary walls, we rule out the prospect that wetting plays a key role in the observed huge difference in water flow rates between graphite and hBN capillaries.

### Effect of surface charge of channel wall’s material

Considering the surface charge as another possible reason, let us recall that capillaries made by mechanical exfoliation have extremely clean surfaces with defect densities and charges of the order of few tens of µC cm^−2^ (MoS_2_ > hBN > graphite)^39^. This is in contrast to nanotubes of the same materials, which are made by chemical vapor deposition and have high surface charge densities of the order of mC cm^−2^, arising from intrinsic defects and contamination during synthesis^7,11^. In nanotubes, there could be pinning of water due to surface defects, but this scenario is not applicable to our 2D channels. To corroborate that surface defects presented on our capillary walls do not impede the observed water flow, we carried out the following experiment. During fabrication, the bottom graphite wall was exposed to oxygen plasma, which created defects and roughened the surface. Oxygen plasma is known to etch graphite in a layer-by-layer manner leaving terraces^40^. Despite the terraced surface of the devices, the water flow was still nearly as fast as in the undamaged channels, without the terraces (Fig. 2c). This indicates that other factors, rather than just atomic flatness, govern the water flow.

### Ionic streaming measurements to study water-wall friction

**Fig. 3:**
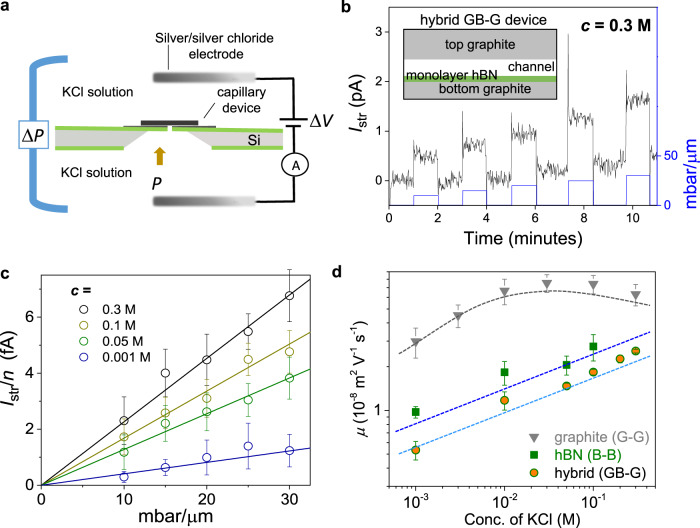
Ion currents driven by applied pressure. **a** Schematics of the experimental setup to measure ionic streaming currents. A known pressure Δ*P* is applied on KCl solution (equimolar on both sides of membrane) from one end and the pressure-induced current was measured by using Ag-AgCl electrodes. **b** Ion streaming currents as a function of applied pressure, for a hybrid device (GB-G) shown in the inset. At a given pressure (shown normalized per 1 µm channel length; blue colored y-axis), *I*_str_ was recorded for one minute. As soon as the pressure is applied the current overshoots sharply and then stabilizes. The KCl concentration here was 0.3 M. **c** At each concentration, *I*_str_ was normalized in the number of channels *n* and plotted against the pressure difference across the channel; the lines represent the best linear fits. Error bars indicate the SD of the measured streaming currents. **d** Streaming (electrokinetic) mobility *µ* for the GB-G device is compared with that found for graphite and hBN devices. GB-G and B-B devices show similar behavior. Dotted curves are guide to the eye. Error bars indicate the uncertainty in the fits of streaming currents vs. pressure.

As the next step, we probed pressure-induced ionic streaming currents, which can provide a measure of the water flow with a full control of the mechanical driving force. In the streaming experiments, ions are transported by water, which is pushed from one end with a known pressure (for details see “Methods”). The pressure-driven ion current (also known as the streaming current, *I*_str_) is sensitive to water-wall friction and can be analyzed using the Poisson–Nernst–Planck (PNP) theory. Recently, some of us reported potassium ion (K^+^) streaming currents using graphite and hBN capillaries, and showed that graphite walls display lower friction for water flow^41^. In the present work, we made a hybrid capillary referred to as GB-G, which had top and bottom graphite layers but with an important difference that bottom graphite was contoured by monolayer hBN. In this device, water and ions were in direct contact with both graphite and hBN. In terms of friction, the PNP theory predicts that large friction at the hBN surface should dominate the behavior of ions and water inside the GB-G capillary^41^. Therefore, if the water-wall friction dominates, one should expect streaming currents to be similar to those for purely hBN capillary devices and they should differ if the electrical properties of surfaces are relevant. With increasing pressure, *I*_str_ was found to increase linearly for various KCl concentrations (Fig. 3). The streaming mobility µ (in the same units as that of electrophoretic mobility) is extracted from slopes of the curves in Fig. 3b. For graphite, hBN and hybrid devices, *µ* are compared in Fig. 3c as a function of KCl concentration. Although *µ* increases linearly for the hBN and GB-G devices, it varies relatively little with the KCl concentration for the graphite device, which is indicative of a low water-wall friction^41^. It is remarkable that, although the walls of the hybrid device were made from graphite, just a monolayer of hBN contouring one of the walls changed *I*_str_ such that the currents became close to those observed in hBN capillaries. This clearly shows the importance of water–hBN interactions.

The K^+^ ion streaming mobility inside graphite capillaries is approximately two to five times higher than that inside hBN capillaries (Fig. 3d), in line with the theory predictions of approximately three to four times lower water friction on graphite in comparison with hBN^21,27^. However, in the evaporation-induced water flow measured by microgravimetry, we have observed a much higher difference (approximately one to two orders of magnitude). Therefore, the difference in water flow rates between hBN and graphite capillaries agrees with theory for our streaming experiments but disagrees for the evaporation ones, despite using essentially the same devices. To understand this conundrum, we recall that there is a major difference between gravimetry and streaming measurements. In the former case, the driving force is evaporation of the so-called extended meniscus. It can spread far away from the capillary mouths and its area effectively determines the driving pressure and, therefore, water flow rates through capillaries^22,42^. If the extended meniscus forms differently on graphite and hBN surfaces, it is likely to alter the rates.

### Fast water permeation due to extended meniscus

To probe the role of extended menisci on graphite and hBN surfaces, we made yet another hybrid capillary device (referred to as BG-B), which had the top hBN wall, whereas the bottom hBN wall was contoured with monolayer graphene (see the schematic shown in the inset of Supplementary Fig. 3). This allowed us to control whether the extended meniscus was on hBN or graphene by mounting the same device differently. If water exited from the side covered with graphene (an extended meniscus could spread along a graphene surface^22,42^), evaporation was much faster (2 × 10^−11^ g s^−1^) compared to the opposite mounting where water was evaporating from an extended meniscus covering hBN surfaces exposed to air (Supplementary Fig. 3). In the latter case, the flow rates were close to those found for purely hBN capillaries without the graphene contouring. This observation suggests that the discussed disagreement was indeed due to a difference in extended menisci on hBN and graphite, which provided a higher driving pressure for the latter capillaries.

### Possible origins of hBN–water interaction

All the described experiments point to the same fact that the reduced water flow observed for hBN capillaries is due to strong interaction of water molecules with the hBN surface. In previous theoretical studies, the enhanced water friction on hBN (as compared to graphite) was attributed to the potential energy landscape created by hBN^27^, electrostatic interaction of hBN with water molecules^21^, and their chemical adsorption^3,43,44^. Although the effect of solid–liquid electrostatic interactions is remarkable on the fluidic properties such as friction coefficient and slip length, it is intriguing to observe that there is a subtle effect on the water contact angle^21^. For a homopolar surface of graphene, the orientation of water dipoles can be perpendicular to the surface^27,45,46^. On the contrary, hBN has a heteropolar surface with a large charge separation between boron and nitrogen atoms^27^. Both experimental and simulation studies have proven that water molecules align their dipoles parallel to the hBN surface^21,27,44^. Furthermore, hydroxide ions (OH^−^) exhibit a strong interaction with nitrogen atoms on the hBN surface^47^; molecular dynamics simulations have shown that the ions can not only be physisorbed but also show stronger chemical interactions with hBN^27,43^. This should suppress the movement of water molecules, resulting in high friction for a water flow inside hBN channels^46,48^. Such strong interaction of water molecules with confining walls is not expected for the case of graphene, thus aiding ultrafast water flow. The water–hBN wall interactions would also likely impede the formation of long-range structurally ordered layers of water, which explains the absence of peak in the water flux through sub-2 nm hBN capillaries or asymmetric capillaries where one wall is made from hBN. For the hybrid capillaries, the underlying surface (graphite in the case of GB-B in Fig. 2c) modifies the water–surface interaction energy landscape of the contoured monolayer hBN, which can explain the mild enhancement observed in the flow compared to B-B capillaries. In the case of MoS_2_, although Mo and S bonds are partially ionic, the large vertical separation between S and Mo atoms make the MoS_2_ surface practically nonpolar with respect to water^49^.

In conclusion, we have examined various factors that can contribute to a large difference in the slip lengths observed for two structurally similar atomically flat surfaces of graphite and hBN. Both our streaming and gravimetry experiments indicate that the graphite surface shows much lower friction (high slip) compared to hBN. The experiments suggest that hBN–water friction arises from electrostatic interaction of polar water molecules with OH^−^ ions adsorbed on the heteropolar hBN surface, which possibly includes the formation of immobile water clusters^50^. In contrast to the prevailing belief that all atomically flat surfaces that are hydrophobic should provide little friction for water flow, our work demonstrates that the friction is mainly governed by electrostatic and similar interactions of flowing molecules with confining surfaces. This understanding is important for the development of nanofluidic channels providing ultrafast flows. For the evaporation-driven technologies such as, e.g., distillation-based separation, our studies show that much higher driving pressures and ultrafast flows could be attained by covering capillary exit surfaces with low-friction graphene layer.

## Methods

### Fabrication of 2D capillary device

The process of making 2D capillary devices is illustrated in Supplementary Fig. 1, with improvements on the previously reported nanofabrication procedure^23^. Graphite and MoS_2_ bulk crystals were obtained from Manchester Nanomaterials. hBN crystals were purchased from HQ Graphene, the Netherlands, with a crystal size of upto 1 mm. All 2D crystals were mechanically exfoliated using scotch tape to expose a fresh crystal on Si wafers with 290 nm thickness of SiO_2_. S1813-positive photoresist, MF319 developer for photolithography, and polymethyl methacrylate (PMMA) resist (molecular weight, 950 K) were purchased from Microposit^®^. Reactive ion etching (RIE) was used for dry etching silicon nitride (SiN_*x*_) and 2D crystals. The μm-size holes in the SiN_*x*_ membrane were etched using RIE with a mixture of SF_6_ and CHF_3_ gases. Graphite was etched using RIE with oxygen gas, whereas hBN was etched in a mixture of CHF_3_ and oxygen gases.

The fabrication starts with a free-standing SiN_*x*_ with dimensions of about 100 μm × 100 μm on a standard silicon (Si) wafer covered with 500 nm-thick layer of SiN_*x*_ on both sides, followed by five parallel hole openings of 3 μm × 30 μm. After that, we transfer a bottom hBN layer on the membrane and back etch the wafer to project the holes in the bottom layer. On a separate ∼290 nm SiO_2_-Si wafer, thin graphene flakes were exfoliated and flakes with thickness ranging from ∼0.7 to 10 nm were identified to serve as spacer layer. These crystals were then patterned by electron-beam lithography using PMMA as a resist and exposed to oxygen plasma to make parallel long stripes of graphite with 130 ± 10 nm wide and 130 ± 10 nm spacing. The residual PMMA mask was removed by mild sonication in acetone and soaking in hot acetone. We transfer this graphene spacer onto the bottom layer and seal the channels with a top hBN layer. After each crystal (bottom, spacer and top) transfer, the SiN_*x*_ wafer chip was annealed in 10% hydrogen-in-argon at 400 °C for 5 h. The annealing steps were essential for the cleanliness of the final devices, to avoid the clogging the channels with PMMA residues and other contaminants from fabrication process.

Hybrid devices (Fig. 2a) were made in a similar method with few additional modifications to the bottom and top layers in the device fabrication flowchart (Supplementary Fig. 1). Monolayers of specific crystals (graphite or hBN as specified in the device name) were transferred onto the bottom crystal before the spacer layer. For instance, in the case of GB-G device, the bottom layer was graphite crystal (~20 nm) covered with a monolayer hBN and the top layer was a graphite crystal (~150 nm). Similarly, the GB-B hybrid device has a top layer made of hBN crystal, whereas bottom is graphite contoured with monolayer hBN, giving rise to both inner capillary walls of hBN surfaces.

### Water flow measurements

Microgravimetry (resolution, 1 µg) was used to investigate the water permeation through 2D capillaries. The SiN_*x*_ chip with 2D capillaries was mounted on an aluminum container filled with deionized water (Millipore Milli-Q) and sealed with chemical-resistant O-rings. The container with chip was then placed on a microbalance (Mettler Toledo XPE26) to monitor the weight loss inside an environmental chamber at a constant temperature (20 ± 0.1 °C) and relative humidity (30 ± 5%).

### Contact-angle measurements

The time-dependent water contact-angle measurements (Supplementary Fig. 5) were performed on the exfoliated 2D crystals, from freshly cleaved (<1 min) through to 4-day ageing. Borosilicate glass capillaries (1.5 mm outer diameter × 0.86 mm inner diameter, Intracel, UK) were pulled (P97 Sutter Puller, UK) to produce pipettes with an opening of <1 µm. These were filled with 6 M LiCl (Sigma Aldrich, UK) prepared using Milli-Q reagent water (Merck Millipore, UK) with a resistivity of 18.2 MΩ cm at 25 °C. LiCl was used to minimize the solvent evaporation, while measuring contact angles^51^. The pipettes were brought into close proximity to the substrate using manual micropositioners with the aid of a charge-coupled device camera (Infinity, Lumenera) and the droplets, diameter of ca. 100 µm, were expelled using a micro-injector (PV820 Pneumatic PicoPump, World Precision Instruments, USA). Contact angles were calculated using a custom-written MATLAB^TM^ script as detailed in an earlier report^51^.

### Streaming current measurements

We used a custom-made electrochemical cell machined from polyether ether ketone for the streaming measurements. It contains two reservoirs sealed with acid-resistant O-rings (James Walker UK, Ltd). Each reservoir can hold up to 2 mL of salt solution and we can insert electrodes (Ag-AgCl) as well, through pressure inlet and outlet pipes (Fig. 3a). The pressure is applied via a microfluidic pump with step size of about 1 mbar (AF1 Dual, Elveflow) and run by using ESI elveflow software interface. After washing the electrochemical cell, O-rings and pressure components were dried with N_2_ gas blow. The devices once mounted on the cell were wet thoroughly with ethanol, ethanol–water (50 : 50), and then deionized water, respectively. Ionic current was measured using Axopatch 200B and Keithley 2636B with LabVIEW software at room temperature (298 K). No significant current was detected from control samples containing no capillaries. After each set of experiments, we unmounted the devices, washed them, and then the experiments were repeated for reproducibility. Streaming current measurements were performed as a function of the applied pressure varying from 0 to 250 mbar. The pressure is applied via the μm-size hole on SiN_*x*_ substrate.

## Supplementary information

Supplementary Information

## Data Availability

All data that support the findings of this study are available within the main text and the Supplementary Materials. Additional data are available from the authors upon reasonable request (see author contributions for specific datasets).
