# Supplementary material for: Water friction in nanofluidic channels made from two-dimensional crystals
Source: Nat Commun. 2021 May 25;12:3092. doi: 10.1038/s41467-021-23325-3 (PMC8149694; doi:10.1038/s41467-021-23325-3)
Supplement: Supplementary file 1 — Supplementary Information [file 41467_2021_23325_MOESM1_ESM.pdf]

Supplementary Information

Water friction in nanofluidic channels made from two-dimensional crystals

Ashok Keerthi [1,2], Solleti Goutham[2,3], Yi You,[2,3] Pawin Iamprasertkun [1,4], Robert A. W. Dryfe [1,2], Andre K. Geim,[2,3] Boya Radha[2,3,*]

[1]Department of Chemistry, University of Manchester, Manchester M13 9PL, United Kingdom

[2]National Graphene Institute, University of Manchester, Manchester M13 9PL, United Kingdom

[3]Department of Physics and Astronomy, University of Manchester, Manchester M13 9PL, United Kingdom

[4]Current address: Department of Applied Physics, Faculty of Sciences and Liberal Arts, Rajamangala University of Technology Isan, Nakhon Ratchasima, 30000, Thailand.

Correspondence to: radha.boya@manchester.ac.uk

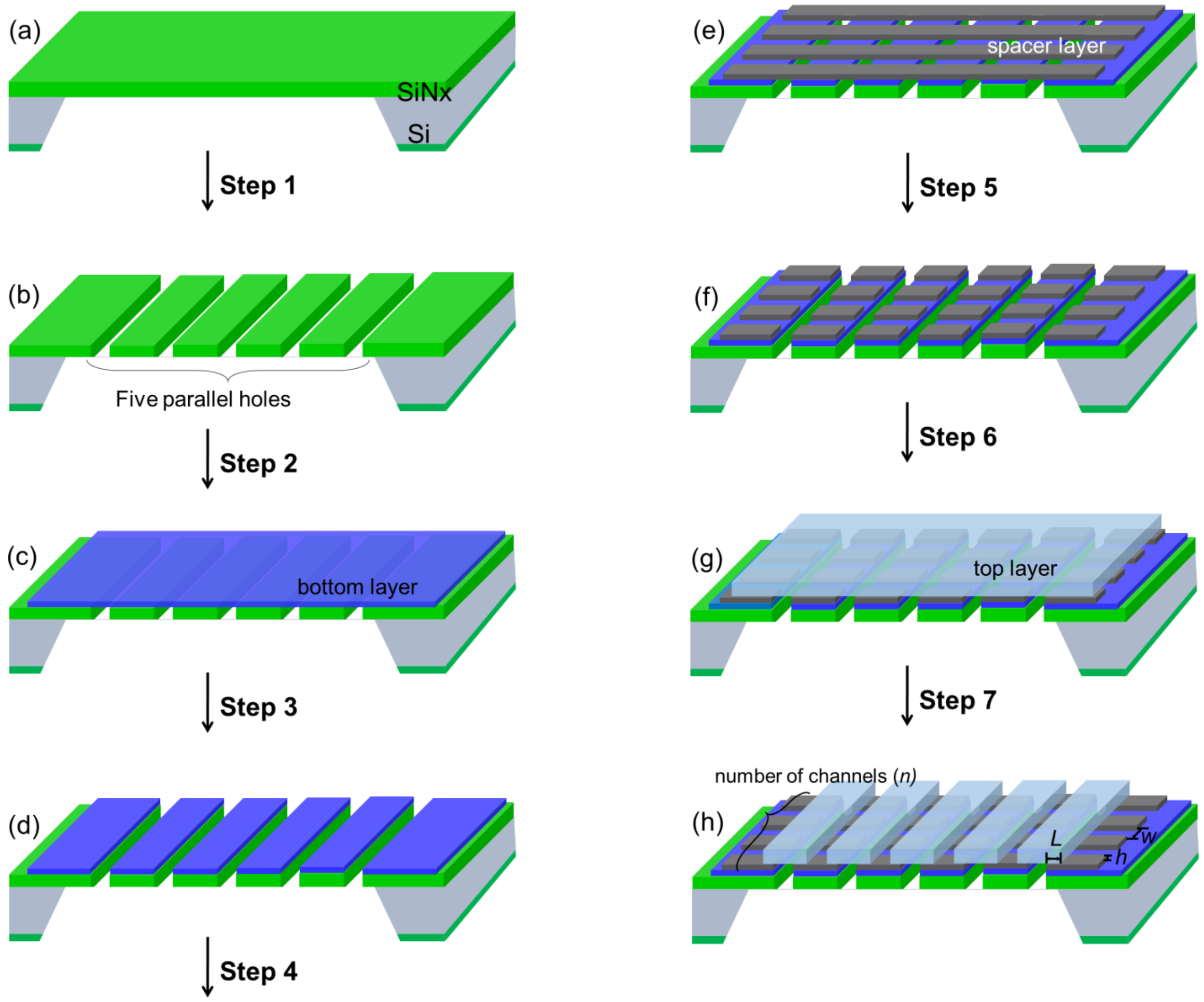

Supplementary Figure 1 | Device fabrication flow-chart for making 2D-capillaries. (a) A free-standing silicon nitride (SiN_x) membrane (shown in green color) supported on silicon wafer (light grey color) with dimensions of about 100 µm × 100 µm made using photolithography and wet etching. (b) In step 1, five parallel rectangular holes (3 µm x 31 µm) with approx. 8 µm spacing were made on the free-standing SiN_x membrane using photolithography and dry etching. (c) In step 2, this membrane with five holes was exposed to oxygen plasma for two minutes to clean the surface. Following this, a mechanically exfoliated thin (thickness, approx. 10 to 20 nm) hBN crystal (shown in blue color) prepared on a separate SiO_2/Si substrate was wet-transferred as a bottom layer, on the SiN_x membrane with five holes. (d) In step 3, the bottom hBN was dry etched (using CHF_3 and O_2) from the backside of SiN_x membrane to project five holes into the bottom hBN crystal. (e) Patterned graphene (dark grey color) stripes prepared on a separate SiO_2/Si substrate, were transferred using wet-transfer method on to the bottom hBN, to serve as spacers. (f) In step 5, these spacer graphite strips were etched from backside of the membrane to remove exposed graphite on the holes area. (g) In step 6, a thick (approx. 200 nm) hBN crystal (light blue color) was transferred on to the stack of bottom and spacer crystals covering all the five holes on SiN_x membrane. (h) In the final fabrication step, the top hBN crystal was patterned using EBL and dry-etched to define the desired channel length (*L*) and opening the capillaries on both sides of each rectangular hole.

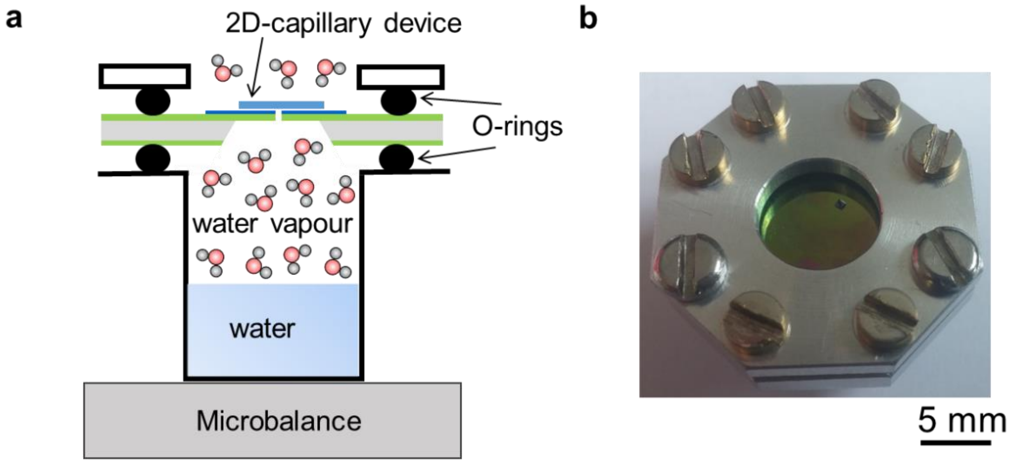

Supplementary Figure 2 | Microgravimetry measurement set-up. a, Schematic representation of microgravimetry measurement setup. **b,** Photograph of the container used to measure water evaporation through 2D channel devices on microbalance.

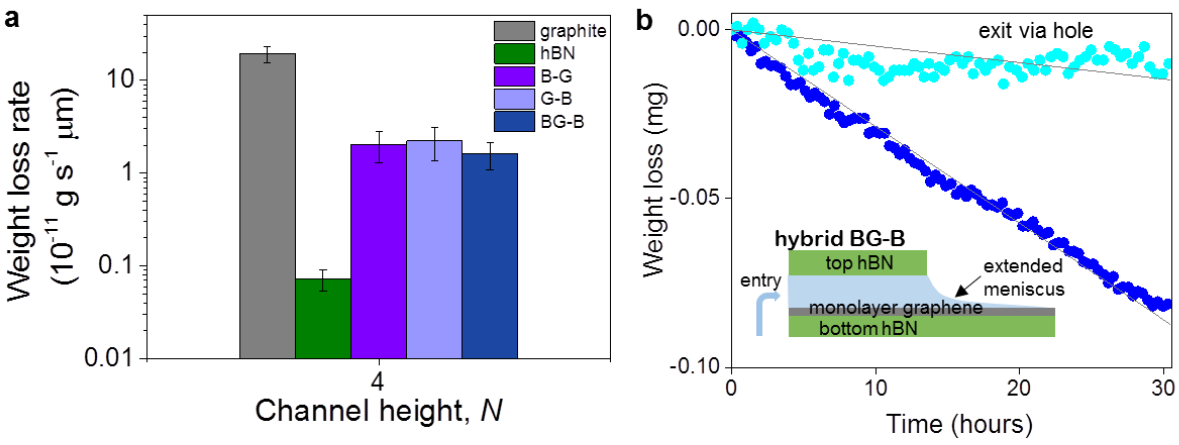

Supplementary Figure 3 | Capillary devices with various 2D surfaces and role of extended meniscus. a, Comparison of gravimetric water flow through graphite channels, hBN channels, asymmetric channels B-G (bottom hBN, top graphite) and G-B (bottom graphite, top hBN), and hybrid channels BG-B (bottom hBN contoured by monolayer graphene, top hBN). All the capillary devices have spacer as 4-layer graphene ($h \approx 1.4$ nm), and the flow is normalized per unit channel length ($L \sim 1$ µm) per channel. Error bars indicate the data scatter for two devices measured for that particular channel. **b,** Water loss through BG-B device shown in a. When the device is mounted such that the entry of water is through the hole and the exit is *via* the channels (blue circles), the extended meniscus forms on graphene layer; bottom inset shows schematic of the extended meniscus on graphene layer. When the sample is mounted in reverse fashion, the exit for water is through the hole (cyan circles), and the extended meniscus forms on hBN edges. The device has 200 channels, with $L \sim 6$ µm. The extended meniscus formed on the graphene monolayer drives the evaporation leads to an increased water flow.

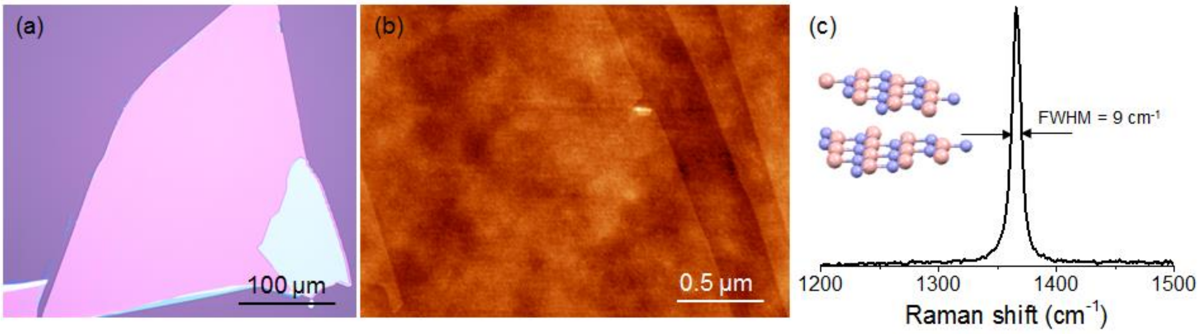

Supplementary Figure 4 | Characterization of freshly exfoliated hBN crystal. a, Optical image of a mechanically exfoliated hBN sheet on SiO_2/Si wafer. **b,** AFM image of a freshly cleaved crystal shows an average roughness of approx. 0.1 nm with typically step heights of 2 nm. **c,** Raman spectrum of hBN thin flake shows a single peak arising from E_{2g} phonon mode at 1366 cm^{-1}, with full width at half maximum of 9 cm^{-1}, which is indicative of the high quality defect free crystal. Inset shows the crystal structure of hBN.

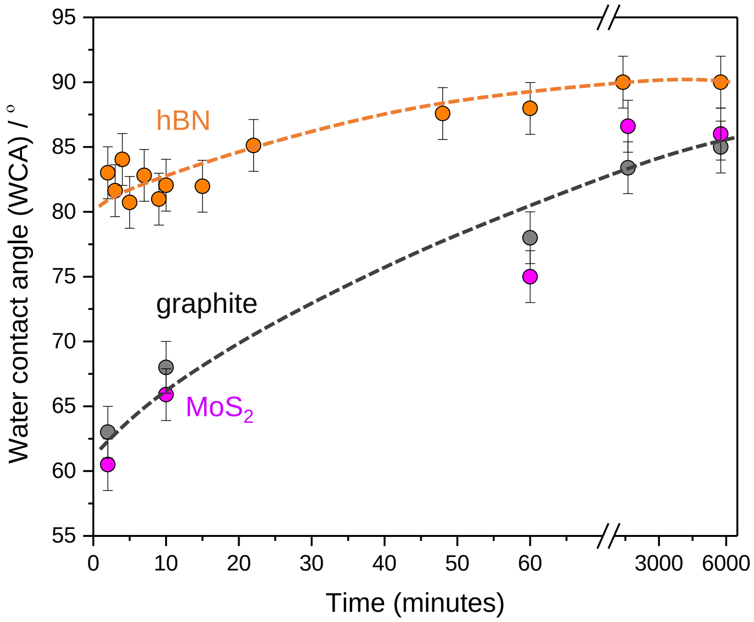

Supplementary Figure 5 | Time-dependent water contact angle measurements on mechanical exfoliated 2D crystals. Contact angles measured on mechanically exfoliated hBN (orange circles), graphite (grey circles) and MoS_2 (pink circles) crystals using 6 M LiCl aqueous solutions at room temperature in ambient conditions. The error bars represent the maximum error in the measurement. Dashed lines are guide to eye.

Supplementary Note 1. Characterizations of 2D materials

Raman measurements were performed to characterize the quality of 2D materials using a micro-Raman setup consisting of an optical microscope, and a spectrograph with 1800 lines/mm grating (Renishaw spectrometer) in a backscattering geometry using a 100X objective lens (numerical aperture ≈ 0.70) under ambient conditions. Atomic force microscopy (AFM) measurements were done with a Bruker Dimension Fastscan instrument using standard silicon nitride tips. The optical image shown in supplementary Fig. 4 shows a freshly exfoliated hBN sheet on a Si/SiO_2 substrate, which is approximately 300 µm in width. AFM analysis on the hBN crystal surface (supplementary Fig. 4) reveals the roughness of the surface to be approx. 0.1 nm with typically step heights of 2 nm. Raman spectra of the hBN sheet shows a single peak arising from E_{2g} phonon mode at 1366 cm^{-1}, with full width at half maximum of 9 cm^{-1}, which is indicative of the high quality defect free crystal.

Supplementary Note 2. Water flow measurements

The channels made from top and bottom graphite walls with channel height N = 4 have shown maximum water flow (data reproduced in Figure 2, from our previous study).[1] The device with 2D-capillaries was usually mounted on the container such that the water enters from the backside of the chip and exit from top 2D crystal side (as shown in inset of Fig. 1c and Supplementary Fig. 2). In the case of vapour-in-contact, water vapour from the container condenses at the entry of the capillary. In water-in-contact experiments, the container was flipped upside down enabling the direct contact of liquid water with the entry of the capillary. Both the experiments yielded similar water flux implying that the water is flowing in the liquid form inside capillaries.

We stored our Å-channel devices in water, to prevent and slow down the hydrocarbon adsorption on the graphitic surfaces.[2] With prolonged storage (typically few weeks to over a month) our Å-channel devices eventually clogged due to the possible contamination of hydrocarbons. The clogged Å-channels can be revived by thermal annealing (400 °C for 5 hours under 10% hydrogen-in-argon). To ensure the cleanliness of the channels in the time-span of the water flow measurement, Helium leak test was done before and after each measurement. When the channels remain clean off residues, the flow of Helium gas is ballistic for ultraflat surfaces of graphene and hBN walls and is quite sensitive to the surface corrugation at an atomic level.[1,3]

The equation (1) relates to the mass flow rate Q through a channel: $Q = \rho_0(h^3/12\eta)(1 + 6\delta/h)Pw/L$, where the ρ_0 is water density; h, w and L is channel height, width and length respectively; η is water viscosity; δ is slip length and P is pressure. This formula assumes long and wide rectangular channels ($L >> w/h$, $w/h >> 1$). For long channels, we can consider the capillary of height h, bound by two long parallel walls as depicted below.

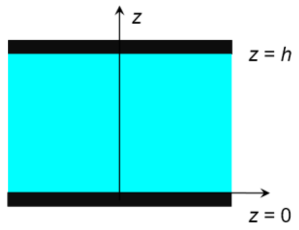

Supplementary Figure 6 | Sketch of channel with height *h*. Two black parallel rectangles represent the bottom and top crystals, respectively. Origin is set at the surface of bottom crystal (i.e. bottom channel wall).

By solving the Navier-Stokes equation for the planar Poiseuille flow, we get

$$\frac{d^2u(z)}{dz^2} = -\frac{P}{\eta L} \qquad \text{(Supplementary Equation 1)}$$

where $u(\mathrm{z})$ is the velocity as a function of position *z*. With no-slip boundary conditions, $u\ (0) = 0, and\ u\ (h) = 0$

The velocity profile for planar Poiseuille flow is given by

$$u\ (z) = \frac{P}{2\eta L} z(h - z) + u_{\mathrm{slip}} \qquad \text{(Supplementary Equation 2)}$$

where u_{slip} is the slip velocity at the boundary walls (*z* = 0 or *z* = *h*), which can be related to the slip length δ as below

$$u_{\mathrm{slip}} = \delta \left| \frac{du(z)}{dz} \right|_{z=0} \qquad \text{(Supplementary Equation 3)}$$

The slip length can be expressed in terms of interfacial friction coefficient λ as $\delta = \eta/\lambda$

Combining the above equations (2) and (3), we get

$$u_{\mathrm{slip}} = \frac{Ph\delta}{2\eta L} \qquad \text{(Supplementary Equation 4)}$$

The liquid flux through the channels is given by

$$Q = \rho_0 w \int_0^h u(z) dz \qquad \text{(Supplementary Equation 5)}$$

From Supplementary equations 2, 4, and 5, we obtain equation (1) given in the main manuscript

$$Q = \rho_0 \frac{P}{12\eta} \frac{w}{L} h^3 \left[1 + \frac{6\delta}{h}\right] \qquad \text{main equation (1)}$$

In nanoscale confinement, when there is large slippage of water flow, i.e., slip length $\delta >> h$, the water slip velocity (at boundary surface) is close to that of the average velocity. One can apply the Poiseuille equation to derive the average velocity, $V = Q/A\rho_0$, where Q is the water flow rate given by equation 1, A is the cross-section area ($A = wh$) and ρ_0 is the water density. For 4-layer thin graphene channels which showed the maximum flow, this yields a flow velocity of approx. 1 m s^{-1}, whereas for hBN channels of same height, the achieved velocity is two orders lower in magnitude, approx. 10^{-2} m s^{-1}.

In addition to the capillary devices with top and bottom walls made from the same crystals (either graphite or hBN), we also made devices where one of the surface is different. Supplementary Fig. 3 shows asymmetric devices where one of the top or bottom is selectively made from either hBN or graphite. The water flux is similar through both asymmetric devices regardless of which of the material was top or bottom layer. To understand the role of extended meniscus, we made hybrid device where the bottom hBN wall was contoured by monolayer graphene and the top was hBN. The extended meniscus formed on the graphene monolayer drives the evaporation and hence leads to an increased water flow.

Supplementary Note 3. Water friction on 2D materials

The polarity of the surface can greatly impact the dipole orientation of (layered / adsorbed) water molecules on the surface.[4-7] In the case of a graphitic surface, water dipole can orient perpendicular to the surface.[5-7] In contrast, hBN has a heteropolar surface due to which the -OH dangling bond will align and approach the nitrogen atom of hBN crystal.[4] This strong intermolecular interaction of water-hBN leads to a high friction flow of water, especially when it comes to the flow of water in the hBN confined channels.[4,5] It has to be noted here that we only discuss the interaction of materials surface with immediate vicinity layer of water molecule. For the bulk water flow, there is no preferred or fixed dipole orientations, instead a wide distribution of the spatial dipole orientations.[8-12]

Supplementary Note 4. Wettability of hBN, graphite, MoS_2 surfaces

We studied the water contact angles (WCA) and the ageing effect on mechanically exfoliated hBN and compared with graphite[13] and MoS_2 crystals.[14,15] In recent years, hBN has gained tremendous attention in 2D materials research due to its extraordinary properties including high temperature and chemical stability, a wide band gap, low dielectric constant and high thermal conductivity, etc.[16-20] Despite remarkable interest, the experimental studies on wettability of water on intrinsic, clean and perfect hBN atomic layer sheets are scarce.[21] Molecular dynamics simulations have provided conflicting WCA on hBN,[22,23] while some predict hBN has similar hydrophobicity to graphite,[23] others report that it is hydrophilic.[22] In our current study, time evolution measurements have shown that airborne contaminants such as hydrocarbons can increase the graphene's intrinsic WCA from about 55° to 85°,[13,24,25] whereas for hBN's intrinsic WCA increased from approx. 80° to 87°.

Supplementary References:

1. Radha, B. *et al.* Molecular transport through capillaries made with atomic-scale precision. *Nature* **538**, 222-225, (2016).

2. Li, Z. *et al.* Water protects graphitic surface from airborne hydrocarbon contamination. *ACS Nano* **10**, 349-359, (2016).

3. Keerthi, A. *et al.* Ballistic molecular transport through two-dimensional channels. *Nature* **558**, 420-424, (2018).

4. Ohto, T., Tada, H. & Nagata, Y. Structure and dynamics of water at water–graphene and water–hexagonal boron-nitride sheet interfaces revealed by ab initio sum-frequency generation spectroscopy. *Phys. Chem. Chem. Phys.* **20**, 12979-12985, (2018).

5. Tocci, G., Joly, L. & Michaelides, A. Friction of water on graphene and hexagonal boron nitride from ab initio methods: Very different slippage despite very similar interface structures. *Nano Lett.* **14**, 6872-6877, (2014).

6. Itoh, H. & Sakuma, H. Dielectric constant of water as a function of separation in a slab geometry: A molecular dynamics study. *J. Chem. Phys.* **142**, 184703, (2015).

7. Brandenburg, J. G. *et al.* Physisorption of water on graphene: Subchemical accuracy from many-body electronic structure methods. *J. Phys. Chem. Lett.* **10**, 358-368, (2019).

8. Grosjean, B., Bocquet, M.-L. & Vuilleumier, R. Versatile electrification of two-dimensional nanomaterials in water. *Nature Comm.* **10**, 1656, (2019).

9. Akaishi, A., Yonemaru, T. & Nakamura, J. Formation of water layers on graphene surfaces. *ACS Omega* **2**, 2184-2190, (2017).

10. Cicero, G. *et al.* Water confined in nanotubes and between graphene sheets: A first principle study. *J. Amer. Chem. Soc.* **130**, 1871-1878, (2008).

11. Dutta, S., Vahdat, M. T., Rezaei, M. & Agrawal, K. V. Crystallization of gas-selective nanoporous graphene by competitive etching and growth: A modeling study. *Sci. Rep.* **9**, 5202, (2019).

12. Ramos-Alvarado, B. Water wettability of graphene and graphite, optimization of solid-liquid interaction force fields, and insights from mean-field modeling. *J. Chem. Phys.* **151**, 114701, (2019).

13. Ounnunkad, K. *et al.* Electrowetting on conductors: Anatomy of the phenomenon. *Farad. Disc.* **199**, 49-61, (2017).

14. Chow, P. K. *et al.* Wetting of mono and few-layered WS_2 and MoS_2 films supported on Si/SiO_2 substrates. *ACS Nano* **9**, 3023-3031, (2015).

15. Heiranian, M., Wu, Y. & Aluru, N. R. Molybdenum disulfide and water interaction parameters. *J. Chem. Phys.* **147**, 104706, (2017).

16. Watanabe, K., Taniguchi, T. & Kanda, H. Direct-bandgap properties and evidence for ultraviolet lasing of hexagonal boron nitride single crystal. *Nat Mater* **3**, 404-409, (2004).

17. Gorbachev, R. V. *et al.* Hunting for monolayer boron nitride: Optical and raman signatures. *Small* **7**, 465-468, (2011).

18. Geim, A. K. & Grigorieva, I. V. Van der waals heterostructures. *Nature* **499**, 419-425, (2013).

19. Dean, C. R. *et al.* Boron nitride substrates for high-quality graphene electronics. *Nature Nanotechnol.* **5**, 722-726, (2010).

20. Withers, F. *et al.* Light-emitting diodes by band-structure engineering in van der waals heterostructures. *Nature Mater.* **14**, 301-306, (2015).

21. Li, X. *et al.* Wettability of supported monolayer hexagonal boron nitride in air. *Adv. Func. Mater.* **27**, 1603181, (2017).

22. Wu, Y., Wagner, L. K. & Aluru, N. R. Hexagonal boron nitride and water interaction parameters. *J. Chem. Phys.* **144**, 164118, (2016).

23. Li, H. & Zeng, X. C. Wetting and interfacial properties of water nanodroplets in contact with graphene and monolayer boron–nitride sheets. *ACS Nano* **6**, 2401-2409, (2012).

24. Kozbial, A. *et al.* Understanding the intrinsic water wettability of graphite. *Carbon* **74**, 218-225, (2014).

25. Li, Z. *et al.* Effect of airborne contaminants on the wettability of supported graphene and graphite. *Nature Mater.* **12**, 925-931, (2013).
